# COVID-19-Associated Pneumomediastinum: An Emerging Clinical Presentation

**DOI:** 10.7759/cureus.18287

**Published:** 2021-09-25

**Authors:** George Hogan

**Affiliations:** 1 Internal Medicine, Eastbourne District General Hospital, Eastbourne, GBR

**Keywords:** surgical emphysema, pneumomediastinum, viral pneumonitis, spontaneous pneumomediastinum (spm), covid 19

## Abstract

Recent publications have suggested an association between coronavirus disease 2019 (COVID-19) pneumonitis and pneumomediastinum. The association has been linked to the frequent use of mechanical ventilation in these patients; however, there have also been increasing reports of spontaneous pneumomediastinum in the absence of mechanical ventilation. These reports suggest a direct association between COVID-19 pneumonitis and increased alveolar fragility. In this report, we present a case of a spontaneous mediastinum in a 64-year-old male patient with COVID-19 without any history of mechanical ventilation.

## Introduction

The recent outbreak of the coronavirus disease 2019 (COVID-19) pandemic has presented clinicians with novel challenges associated with the diagnosis and optimal management of this new disease. Among the uncommon manifestations of coronavirus infection is the development of pneumomediastinum, normally a rare condition associated with mechanical ventilation, existing lung disease, or blunt force trauma [1]. Such cases have been reported in mechanically ventilated patients, but also as sequelae of COVID-19 infection even in the absence of ventilation [2]. The pathophysiology of this condition remains obscure. However, some reports suggest that despite the clinicians' concern, it can be a benign finding, with spontaneous resolution being the rule [2].

## Case presentation

A 64-year-old man presented to the hospital with a four-day history of worsening shortness of breath associated with fever and dry cough. He had been previously very fit and well and was still working as a mechanic. His only past medical history involved well-controlled asthma, managed with beclomethasone and salbutamol inhalers. Initial examination revealed mildly increased work of breathing and scattered crepitations in the left base of the chest.

**Figure 1:**
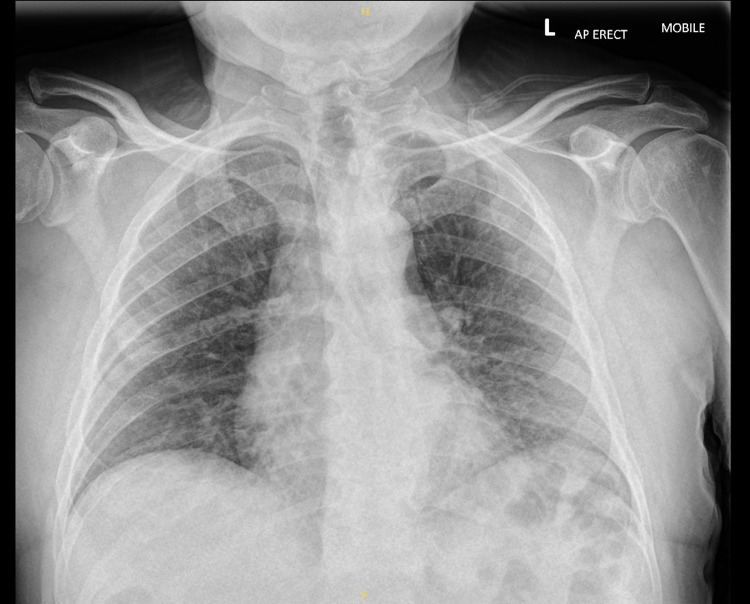
Admission chest X-ray demonstrating only mildly increased lung markings

COVID-19 infection was strongly suspected given the history and confirmed with viral polymerase chain reaction sampling. The patient was started on treatment with dexamethasone, salbutamol and ipratropium nebulizers, and oral antibiotics for possible superimposed bacterial infection. He was also started on 35% inspired oxygen therapy to maintain target oxygen saturations greater than 94%. Chest X-ray performed on the day of admission showed mild ground-glass changes bilaterally, typical of COVID-19 pneumonitis, but no other findings (Figure 1).

**Figure 2:**
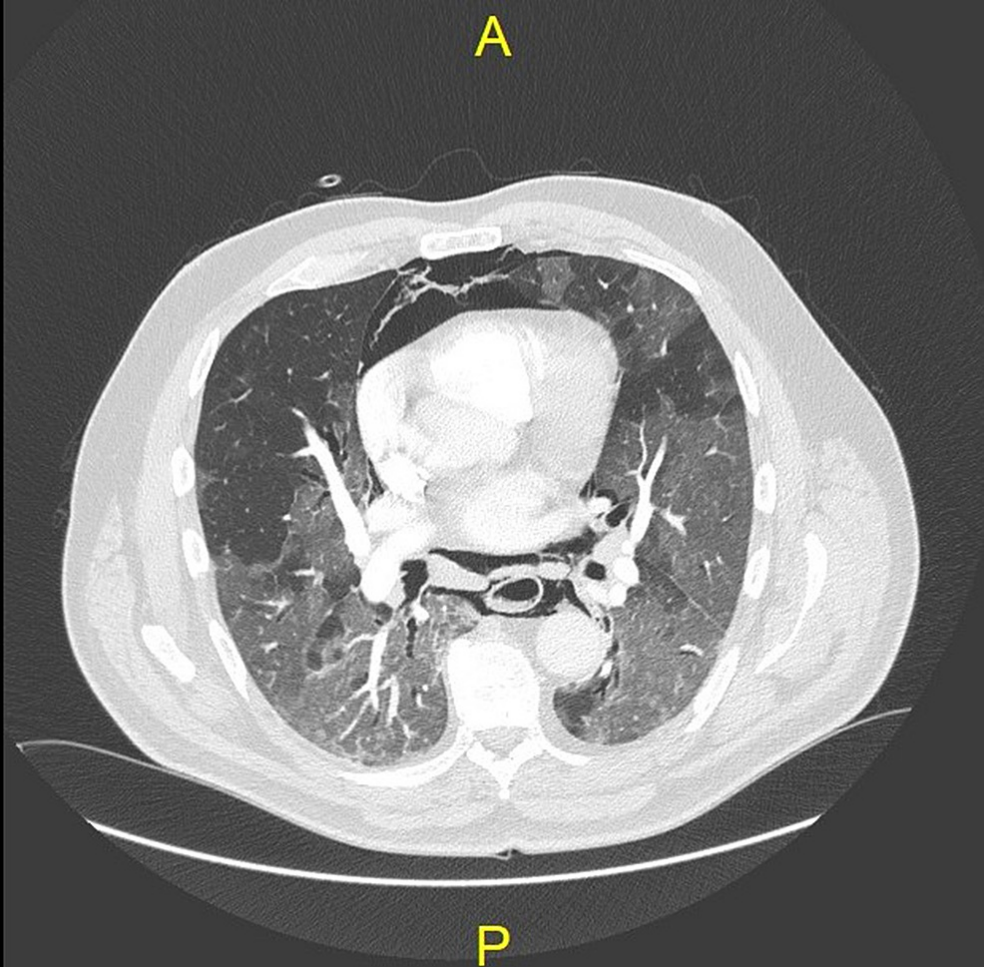
Coronal CTPA slice demonstrating extensive pneumomediastinum and associated COVID-19 pneumonitis CTPA: computed tomography pulmonary angiogram; COVID-19: coronavirus disease 2019

**Figure 3:**
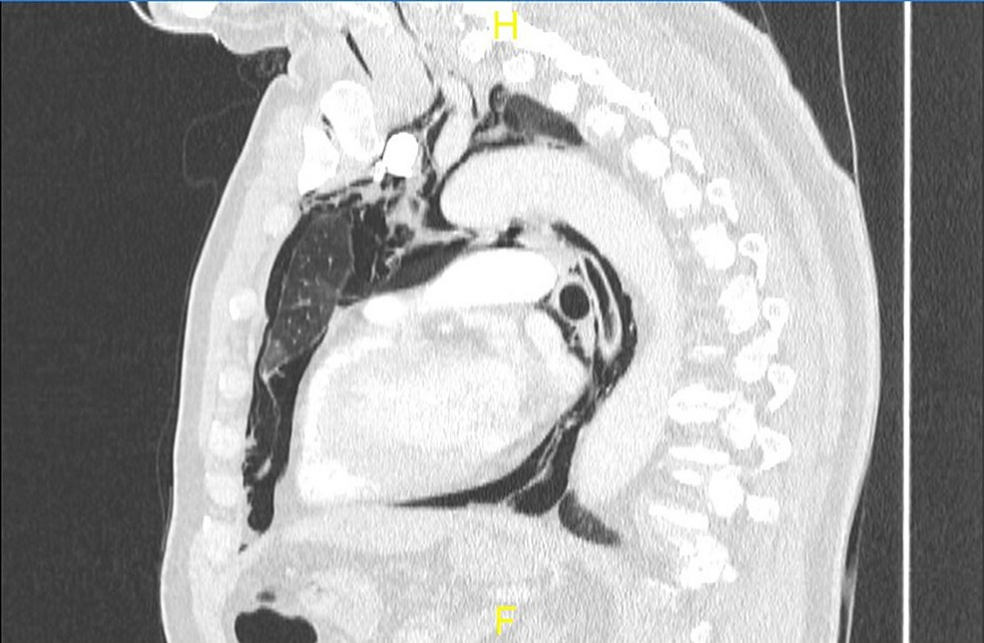
Sagittal CTPA slice demonstrating pneumomediastinum CTPA: computed tomography pulmonary angiogram

The patient remained clinically stable but underwent CT pulmonary angiography (CTPA) the following day due to a raised D-dimer level, revealed in a test performed as part of the initial admission blood sampling. No evidence of pulmonary embolism was found, but imaging revealed an unexpected incidental large pneumomediastinum as well as diffuse changes typical of COVID-19 pneumonitis (Figures 2, 3). Widespread surgical emphysema was also noted on the imaging.

These changes had not been present on the chest X-ray the previous day. The patient initially continued to feel well despite his oxygen demand; examination following the scan results revealed surgical emphysema in the soft tissues of the neck but no other abnormalities.

**Figure 4:**
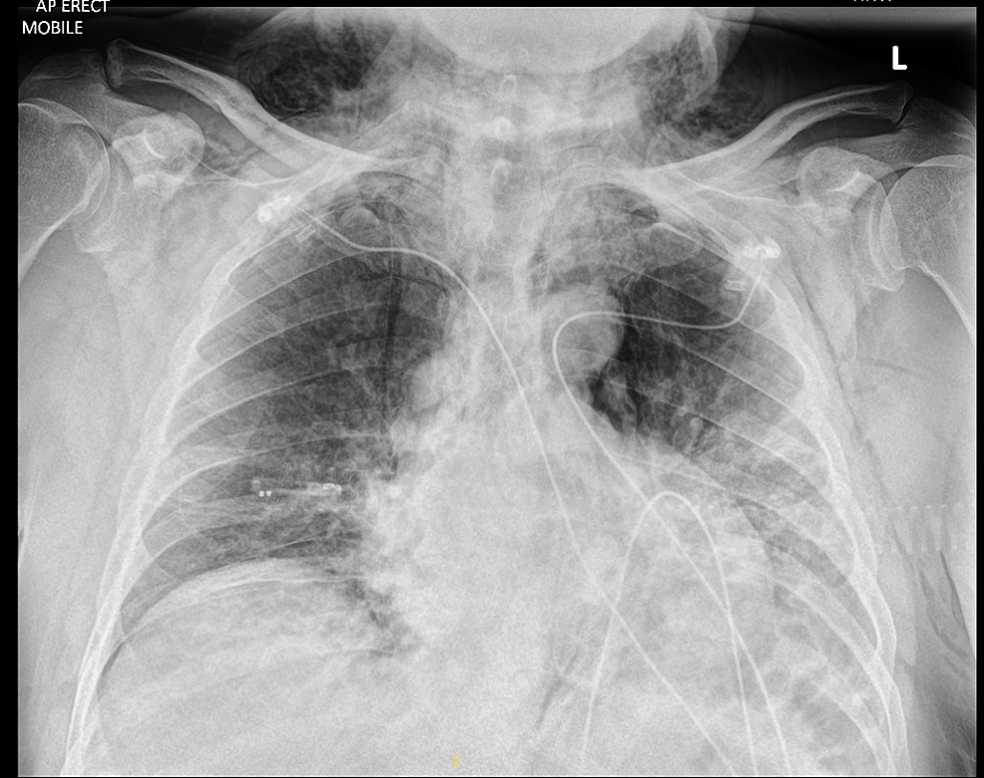
Repeat chest X-ray following CTPA demonstrating the progression of viral pneumonitis and surgical emphysema CTPA: computed tomography pulmonary angiogram

Shortly after, the patient began to deteriorate with rapidly increasing oxygen demand to 100% Fi0_2_. Repeat chest X-ray did not show any evidence of pneumothorax associated with the pneumomediastinum, but there was a significant progression of the viral pneumonitis seen on the previous scan (Figure 4). Blood gas analysis was performed, which showed pO_2_ of 9.3 kPa on high-flow oxygen, and the patient was rapidly shifted to the intensive care unit (ICU) for ventilatory support. His case was discussed with cardiothoracic surgeons following the incidental finding, and they advised conservative management of the pneumomediastinum in the absence of evidence of extension from a pneumothorax, and intubation rather than non-invasive ventilation should respiratory support be required. While in ICU, the patient was offered tocilizumab as part of the RECOVERY trial to which he consented.

Following a two-day stay in the ICU, the patient's oxygen demand gradually declined. He was stepped down to a level 1 ward on 4 liters of oxygen through nasal cannulae as he continued to maintain his saturations. The patient reported a subjective reduction in the surgical emphysema in the tissues of his neck. On discussing the events, he revealed that shortly before the CTPA scan was performed, he had felt a subjective “bursting” sensation in his chest, which he had associated with the development of the pneumomediastinum. The patient has now fully recovered and has been discharged from the hospital.

## Discussion

Pneumomediastinum is a rare condition, more commonly seen in male patients, which is characterized by an abnormal presence of air in the mediastinum. It is generally associated with blunt force trauma to the chest, forceful vomiting, and barotrauma secondary to mechanical ventilation [3]. Spontaneous pneumomediastinum is rarer, and it is associated with preexisting lung diseases such as asthma, and smoking [3].

More recently, reports have suggested an association between COVID-19 pneumonitis and pneumomediastinum [2,4,5]. Some authors have linked this association to the high levels of mechanical ventilation used in patients with severe COVID-19 disease. Patients with COVID-19 have a significantly elevated risk of pneumomediastinum and pneumothorax; the incidence of pneumothorax in mechanically ventilated patients with COVID-19 has been estimated to be 13% [6]. However, an analysis that compared cohorts of COVID-19 patients with non-COVID-19 patients on ventilation showed that patients with COVID-19 have a significantly higher incidence of pneumomediastinum compared to other ventilated patients with acute respiratory distress syndrome (13.6% vs. 1.9%), suggesting that coronavirus infection may have a causal role [7].

There have been several reports of spontaneous pneumomediastinum in patients with COVID-19 even without mechanical ventilation, as in this case, and a similar association has previously been noted in severe acute respiratory syndrome (SARS) pneumonitis [8]. The mechanism is not clearly understood, although a diffuse alveolar injury is common in COVID-19 patients, as is frequent coughing leading to raised intrathoracic pressure. This may increase the chance of alveolar rupture with the gas flow along the sheath of the bronchi and pulmonary vessels towards the mediastinum, a phenomenon known as the Macklin effect. Despite the increased medical concern caused by a diagnosis of pneumomediastinum associated with COVID-19 pneumonitis, most cases can be managed conservatively with a good outcome and gradual resorption of air back into tissues [9]. However, further investigations may be warranted to rule out pneumothorax in these patients, which is clearly associated with worse outcomes [6].

## Conclusions

A clear association has been demonstrated between COVID-19 and pneumomediastinum but it remains unclear if this linkage is related to the increased use of mechanical ventilation in these patients. A growing body of case reports and series suggests that at least part of this association is due to the direct effect of COVID-19 on lung parenchymal fragility. This case adds to the small but growing body of literature highlighting this association between COVID-19 and pneumomediastinum, even in the absence of ventilatory support.

## References

[1] Kouritas VK, Papagiannopoulos K, Lazaridis G (2015). Pneumomediastinum. J Thorac Dis.

[2] Mohan V, Tauseen RA (2020). Spontaneous pneumomediastinum in COVID-19. BMJ Case Rep.

[3] Caceres M, Ali SZ, Braud R, Weiman D, Garrett HE Jr (2008). Spontaneous pneumomediastinum: a comparative study and review of the literature. Ann Thorac Surg.

[4] Sun R, Liu H, Wang X (2020). Mediastinal emphysema, giant bulla, and pneumothorax developed during the course of COVID-19 pneumonia. Korean J Radiol.

[5] Wang J, Su X, Zhang T, Zheng C (2020). Spontaneous pneumomediastinum: a probable unusual complication of coronavirus disease 2019 (COVID-19) pneumonia. Korean J Radiol.

[6] Chopra A, Al-Tarbsheh AH, Shah NJ (2021). Pneumothorax in critically ill patients with COVID-19 infection: incidence, clinical characteristics and outcomes in a case control multicenter study. Respir Med.

[7] Lemmers DH, Abu Hilal M, Bnà C (2020). Pneumomediastinum and subcutaneous emphysema in COVID-19: barotrauma or lung frailty?. ERJ Open Res.

[8] Chu CM, Leung YY, Hui JY (2004). Spontaneous pneumomediastinum in patients with severe acute respiratory syndrome. Eur Respir J.

[9] Volpi S, Ali JM, Suleman A, Ahmed RN (2020). Pneumomediastinum in COVID-19 patients: a case series of a rare complication. Eur J Cardiothorac Surg.

